# Optimising care for adults with learning disabilities with a diabetes diagnosis: a commentary

**DOI:** 10.1108/TLDR-02-2026-0011

**Published:** 2026-05-18

**Authors:** E Hodgson, L Robson, J Harrison, A Doherty

**Affiliations:** Lancaster Medical Practice; The Pendle Medical Partnership Colne Corner Surgery; University of Lancashire

## Abstract

**Purpose:**

People with a learning disability are at a higher risk of developing diabetes than the general population. For them, and for those that care for them, a diagnosis of diabetes is associated with challenges. This paper explores the issues involved in optimising care for people with a learning disability to both prevent and manage diabetes in this population effectively and equitably. The paper is designed to inform clinical practice, improvements in patient care, and further research in this field.

**Design/Methodology/Approach:**

A commentary which summarises and critically appraises a systematic review by Holden & Lee (2022) that examined the barriers and facilitators involved in optimal diabetes care for people with a learning disability. The commentary expands upon the review’s findings in the context of clinical practice.

**Findings:**

The original systematic review identified twelve main barriers and fourteen main facilitators to optimal diabetes care for people with a learning disability at different individual, relationship, community and societal levels.

**Originality:**

The authors of this commentary argue that strengthening multidisciplinary collaboration, providing education, reasonable adjustments, tailored interventions and improved communications, enhances self-management and ensures the optimisation of person-centred, equitable diabetes care for people with a learning disability across all healthcare practice settings.

## Introduction

The prevalence of diabetes is increasing; it is estimated that more than 5.8 million people are living with diabetes in the UK (Diabetes UK, 2025). The odds of having a diagnosis of diabetes are 2.5 times higher in people with a learning disability compared to the general population (Vancampfort *et al*., 2022). Additionally, the increase in rates of type two diabetes is around 10-15 years earlier for people with a learning disability compared to the general population (Baksh *et al*., 2023).

For people with a learning disability, a diagnosis of diabetes is associated with several challenges including a lack of diabetes awareness and healthy lifestyle, reliance on family or carers for support with diabetes management, less access to supportive education programmes, and lack of appropriate training for care providers (Maine *et al*., 2020; Taggart *et al*., 2014). People with a learning disability have a wide variety of complex communication needs which can have serious implications if not appropriately managed by those responsible for their healthcare needs (Patel *et al*., 2018). In addition, people with a learning disability and diabetes receive less proactive monitoring such as blood tests, or eye and foot examinations (Baksh *et al*., 2025). Macrovascular complications following a diagnosis of diabetes (such as heart disease and stoke) are more likely for those with a learning disability (Baksh *et al*., 2025), and the risk of diabetes related mortality is doubled (Wing and Mathur, 2025).

There is a need for improved diabetes care and education for individuals with a learning disability to prevent and manage diabetes effectively and equitably (Baksh *et al*., 2023). Supported self-management, person-centred care, and reasonable adjustments to education are crucial to reducing inequalities for people with a learning disability and a diagnosis of diabetes (Beresford and Kozlowska, 2022). In response to the poorer health outcomes and barriers to care experienced by people with a learning disability, the Health and Social Care Act (2022) requires mandatory training on learning disability for all health and care providers (Department of Health and Social Care, 2022). The Oliver McGowan Code of Practice sets out standards for the mandatory training, emphasising safe, equitable and person-centred care (Department of Health and Social Care, 2025a). To support these standards, it is crucial to understand the difficulties experienced by people with a learning disability and a diabetes diagnosis. This commentary critically appraises and explores the findings from a systematic review which identifies the barriers and facilitators involved in optimal diabetes care for people with a learning disability, published by Holden and Lee (2022).

### Critical Appraisal of Holden and Lee (2022)

Using the Joanna Briggs Institute (JBI) critical appraisal checklist tool for systematic reviews (JBI, 2017), the review achieved 9 out of the 11 criteria (Table 1). The main strengths of the review included a clear research question / inclusion criterion, and a robust search strategy. A narrative synthesis was appropriately applied to combine the results of the included studies with themes checked by two reviewers, and there were clear recommendations for policy, practice and future research. It was not clear however if data extraction or quality assessment were undertaken independently by two reviewers. This increases the likelihood of errors from individual judgements and reduces the reliability of the results (Li *et al*. 2019). Overall, the review provides a comprehensive overview of the current evidence base for barriers and facilitators to optimising diabetes management for adults with learning disabilities. Some caution should be applied however to the interpretation of results due to the limitation identified.

## Results of the systematic review by Holden and Lee 2022

Following identification of 6255 articles through the database search, 33 articles were reviewed in full, resulting in eight selected for inclusion. A further two articles were identified from hand searching resulting in ten included studies overall. Of the ten included articles, eight were primary research studies and two were guidance documents outlining reasonable adjustments for people with learning disabilities. Research settings varied, but all were based in high income countries and included learning disability services and residential locations. Of the research studies, three were appraised to be of high quality, three of medium quality, and two of low quality. The two guidance documents were not quality assessed as they did not contain qualitative findings.

### Barriers identified by the review

Twelve unique barriers to optimal diabetes care in those with learning disabilities were identified and categorised within four levels of a social-ecological model: the individual, relationship, community and societal. Most of the barriers were identified as high or medium reliability. At the individual level, barriers were a low level of diabetes knowledge and having multiple other health conditions. Relationship-related barriers included a lack of diabetes knowledge from caregivers, and relationships with caregivers who discourage independent autonomy. Community barriers related to lack of self-management opportunities in communal living arrangements, independent living (the only barrier identified as low reliability), and experiencing feelings of stigma. Societal barriers include: a lack of accessible information, systems incompatible with making reasonable adjustments, compromise on ideal diabetes management, inappropriate self-management programmes, and inconsistent care plans. The most reported barrier identified was low level of diabetes knowledge and understanding for the individual with a learning disability.

### Facilitators identified by the review

Fourteen unique facilitators were identified, most of these were of high or medium reliability, except for two of low reliability, and two that were not assessed for reliability. Facilitators to optimal diabetes care at the individual level include motivation to self-manage the condition and a high level of diabetes knowledge and understanding. At the relationship level, facilitators include the presence of caregivers, close relatives with diabetes, peer support, and autonomy facilitated by a caregiver. A consistent approach from caregivers was also identified but was of low reliability. At the community level, living in residential care and addressing social barriers were identified as facilitators, yet these were of low or unassessed reliability. At the societal level, person-centred planning and reasonable adjustments are facilitators, as well as adapted support programmes, collaborative approaches, accessible information, and training for staff and caregivers. The most reported facilitator was ‘person-centred planning’ and ‘reasonable adjustments.’ Table 2 summarises the identified key barriers and facilitators identified by Holden & Lee (2022).

## Commentary

The findings from Holden and Lee (2022) indicate that a lack of diabetes knowledge or understanding for the individual or the caregiver, is a common barrier to optimal diabetes care. A lack of reasonable adjustments within healthcare systems, reduced opportunities for self-management, and a lack of appropriate care plans provide further challenges to equitable care. By contrast, a high level of diabetes understanding and motivation to self-manage at an individual level can help to overcome these challenges. Person-centred care, adequate training for formal and informal caregivers, reasonable adjustments, and caregiver support are further enablers to optimal care.

### Diabetes education and reasonable adjustments are vital for the care of people with a learning disability and a diabetes diagnosis

Improving understanding of diabetes at an individual and carer level is fundamental (Holden and Lee (2022) yet people with a learning disability and diabetes have limited access to educational resources and to healthcare professionals trained in learning disability communication skills (Taggart *et al*., 2014). Such factors may contribute to suboptimal care and an increased risk of complications (MacRae *et al*., 2015). Structured education to improve health literacy and diabetes management in people with a learning disability is required, together with education and training for carers (Maine *et al*., 2020). DESMOND-ID is a diabetes educational programme specially adapted for people with a learning disability which aims to encourage lifestyle improvements and self-management (Chatterjee *et al*., 2018). Unfortunately, however, individuals requiring resources such as personal budgets needed to pay for caregiver support may experience challenges in accessing such educational enablers (Micai *et al*., 2022). Diabetes UK (2022a) provides free resources on their website to improve care for people with diabetes and a learning disability (see Table 2).

Holden and Lee (2022) identified reasonable adjustments as an enabler to optimal care. People with a learning disability have a legal right to reasonable adjustments when accessing health and social care to ensure that they are not disadvantaged compared to their non-disabled peers (Equality Act 2010; Department of Health and Social Care 2022). The `NHS Right Care Pathway for Diabetes’ (NHS Right Care, 2017) further highlights the importance of making reasonable adjustments for people with a learning disability who have diabetes, and the associated benefits e.g. fewer visits to the GP, fewer diabetes related complications and hospital admissions. Health and social care professionals must be trained to work collaboratively, flexibly, and in a person-centred manner to ensure that reasonable adjustments are made for this population group (Beresford, 2022). Box 1 highlights examples of reasonable adjustments which can be made to help people with a learning disability who manage their diabetes with insulin. These examples are adapted from Beresford (2022), the NHS Right Care Pathway for Diabetes (NHS Right Care, 2017); and Diabetes UK (2018) and informed by people who have diabetes and a learning disability, and through discussions involving their families, carers and health care professionals.

### Person-centred care and caregiver involvement as foundations for effective diabetes self-management

Person centred planning, the presence of a caregiver and self-management facilitated by a caregiver were identified as key enablers to the best possible care (Holden and Lee, 2022). Person-centred approaches such as treating people as individuals and making reasonable adjustments, are key requirements for diabetes self-management in adults with learning disabilities (Beresford and Kozlowska, 2022). Development of networks between primary healthcare and specialist learning disability services may lead to improvements in communication, information sharing, and safer, person-centred care (Brown *et al*., 2017). The international consensus guidelines for managing type 2 diabetes in people with a learning disability recommends liaison between primary care and specialist diabetes services including shared protocols and the involvement of carers (Taggart *et al*., 2021). Involving Speech & Language Therapists in such professional networks may help improve services’ understanding of the communication needs of people with a learning disability and improve resources for those who have diabetes (Lewer and Harding, 2013).

Self-management interventions are feasible for adults with learning disabilities and yield positive outcomes (Devitt *et al*., 2025). Self-management does not mean that people with a learning disability must manage their healthcare alone. Self-management support from carers helps give patients the level of support needed so that they can manage their chronic conditions daily and thus help develop their confidence to sustain healthy behaviours (Cardol *et al*., 2012). Caregiver support however is mostly directed to administering medication and controlling food intake; more education for caregivers about what comprises person-centred care and self-management in people with a learning disability who have diabetes is needed (Cardol *et al*., 2012). Gordon-Brown *et al*., (2025) explored peer support amongst family carers of people with a learning disability, demonstrating that shared experiences and mutual support enhance carers’ confidence and coping strategies, thus highlighting the value of community-based learning opportunities in empowering caregivers.

### Addressing health inequality and advancing diabetes care for people with a learning disability

The barriers highlighted by Holden and Lee (2022) highlight many of the health inequalities experienced by people with a learning disability and a diagnosis of diabetes. The NHS 10-Year Long Term Health Plan (DHSC, 2025b) recognises that people with a learning disability experience greater health inequality compared to the general population, including a lower life expectancy and higher rates of avoidable deaths. The Plan recommends reasonable adjustments [*a flexible approach to care, resources and education that are adapted to the needs of people with learning disabilities]* (Beresford and Kozlowska 2022; NHS England 2023a); improved access and specialist support; community-based care; and accountability (DHSC, 2025b). Diabetes is a clinical priority within the Plan including greater support, expansion of the NHS Diabetes Prevention Programme (NHS England, 2025) and improved equity of access. The National Institute for Health and Social Care Excellence (NICE) is committing to re-evaluating clinical pathways described in the 10-year Health Plan by shifting from a `one size fits all’ approach to personalised treatment plans, including access to newer diabetes medicines (NICE, 2025a).

Although the 10-Year NHS Plan commits to more holistic ongoing care and the expansion of technologies, there are no known tailored technological measures for people with learning disability who have diabetes. A study by Álvarez-Aguado *et al*., (2025) explored the experiences of older adults with learning disabilities in using technology. The research identified challenges such as complex interfaces and cognitive limitations but also emphasized the empowering role of technology in fostering independence and social connections. Caregivers were found to be critical in facilitating technological adoption, underscoring the need for targeted training programs to enhance caregivers' technological skills. The use of advancing technology is showing excellent results for diabetes patients in paediatrics and the general population (Dos Santos *et al*., 2021; Stamati *et al*., 2022). Considering the direction of diabetes care around continuous glucose monitoring and insulin pumps to achieve optimum control, the need for staffing resources to ensure those with learning disabilities can access this technology is further underlined (NICE 2025b). Future research could usefully explore the use of advanced technology in diabetes management involving those with learning disabilities compared to those without learning disabilities.

### Diabetes related research and inclusiveness of people with a learning disability

Holden and Lee (2022) discuss the need for further rigorous research in optimal diabetes care with people that have a wide range of learning disability. Taggart *et al*., (2024) suggest that barriers to the inclusion of people with a learning disability in diabetes specific research have tended to revolve around factors relating to the individual, research design and methodologies, and the system and / or organisation. People with a learning disability may experience difficulties in understanding the amount, type, and level of involvement in research expected (Strnadová and Cumming, 2014). People with a learning disability also experience high levels of social deprivation (Emerson, 2013) and social exclusion (Pownall *et al*., 2020) which can affect the ability of some to access research opportunities. Diabetes related research must be inclusive and focus on how individuals with a learning disability can be supported to manage their diabetes – some of whom may not be able to participate in more conventional research such as verbal interviews (Beresford, 2022). The DESMOND-ID Study importantly highlights the types of reasonable adjustments that can be made to involve people with a learning disability in diabetes related research and education: My Diabetes and Me Study - DESMOND-ID

## Conclusions

The review by Holden and Lee (2022) identified several challenges to optimal diabetes care for people with learning disability and emphasised the need for services to provide interdisciplinary, person-centred support. Positive influences on diabetes management include reasonable adjustments, supportive carers, accessible education, and the use of adapted programmes for people with learning disabilities such as DESMOND-ID. These findings align with the aims of the NHS Long Term Plan (DHSC, 2025) to reduce health inequalities and promote equitable high-quality care. However, despite these commitments, progress remains inconsistent, and people with learning disabilities continue to experience barriers to accessing and utilising effective diabetes care management. Strengthening multidisciplinary collaboration, effectively responding to the communication needs and styles of people with a learning disability, providing education, reasonable adjustments, and involving people with a learning disability in diabetes research are all essential in the development of tailored interventions, enhancing self-management and ensuring that person-centred and equitable diabetes care becomes standard across all healthcare settings.

## Figures and Tables

**Table 1:** Critical appraisal of the review by Holden and Lee (2022) using the JBI critical appraisal checklist for systematic reviews and research syntheses (JBI, 2017)

JBI critical appraisal checklist items	Responses
1. Is the review question clearly and explicitly stated?	Yes, the Population Interest and Evaluation (PIE) study design framework was used to formulate the review question: to review relevant literature to identify barriers and enablers to optimal diabetes care for adults with learning disabilities.
2. Were the inclusion criteria appropriate for the review question?	Yes, inclusion criteria were literature related to adults with a learning disability, and caregivers of individuals with a learning disability. Any type of publication and all study designs with a qualitative aspect were included. Articles with no reference to diabetes management, the learning disability population, or enablers/barriers to care were excluded. Non-English articles were also excluded due to time constraints.
3. Was the search strategy appropriate?	Yes, the PIE framework was used to formulate search terms. Synonyms for key terms were obtained from a previous review with a similar population.
4. Were the sources and resources used to search for studies adequate?	Yes, six electronic databases including those from medical, psychological and social science domains were searched from inception to August 2019. This included a grey literature database.
5. Were the criteria for appraising studies appropriate?	Yes, critical appraisal was undertaken using an adapted version of the Critical Appraisal Skills Programme (CASP) Tool for qualitative research.
6. Was critical appraisal conducted by two or more reviewers independently?	No, the review does not state how many reviewers conducted critical appraisal.
7. Were there methods to minimize errors in data extraction?	No, the review does not state how many reviewers conducted data extraction, only that the themes were double-checked by a second reviewer. No reference to any tools used to guide data extraction.
8. Were the methods used to combine studies appropriate?	Yes, themes were evaluated through narrative synthesis and applied to the social-ecological model.
9. Was the likelihood of publication bias assessed?	Non-Applicable to systematic reviews of qualitative evidence.
10. Were recommendations for policy and/or practice supported by the reported data?	Yes, recommendations for commissioners and service providers include structured education programmes for use by the learning disability population with diabetes, uptake of person-centred reasonable adjustments in healthcare systems, accessible information provision, and educational programmes for healthcare professionals/caregivers.
11. Were the specific directives for new research appropriate?	Yes, reviewers recommend future rigorous research with adults that have a wide range of learning disability (mild-severe). The effectiveness of adapted educational programmes should also be assessed.

**Table 2:** Barriers and facilitators to optimising diabetes care for people with a learning disability identified by Holden & Lee (2022)

Barriers	Facilitators
**Individual level**	
Low level of diabetes knowledge and understanding Multiple other health conditions	Motivation to self-manage condition High level of diabetes knowledge and understanding
**Relationship level**	
3. Formal or informal caregivers lack diabetes knowledge and understanding 4. Relationships with carers who do not encourage individual autonomy	3. Presence of formal or informal caregivers 4. Close relatives with diabetes 5. Peer support 6. Autonomy facilitated by caregiver 7. Consistent approach from caregivers
**Community level**	
5. Communal living arrangements 6. Independent living 7. Stigma	8. Living in residential care 9. Social barriers addressed
**Societal level**	
8. Lack of accessible information 9. Systems do not allow reasonable adjustments 10. Compromise on ideal diabetes management 11. Inappropriate structured self-management programmes 12. Inconsistent and disjointed care plans	10. Person-centred planning and reasonable adjustments 11. Adapted support programmes 12. Collaborative approaches 13. Accessible information 14. Training for staff and caregivers

## Box 1 Reasonable adjustments for supporting people with a learning disability to manage their diabetes

**Support for diabetes appointments** – e.g. check to see if the person with a learning disability wishes to have a carer involved in their healthcare and if carer support is available, if required

**Flexible appointments** – e.g. to fit around the person and carers who support them.

They may require longer or extra appointments

**Use simple language** and avoid jargon – e.g. use simplified language, assess whether they understand questions asked / information provided

**Make information accessible** – to help people understand. For example: provide accessible resources for individuals with a learning disability to (a) identify risk factors and prevent diabetes; and (b) for the diabetes self-management e.g., Diabetes UK (2022a) resources for people with diabetes and a learning disability

**Use videos** – e.g. to support the person with insulin injections, and glucose monitoring.

**Provide training** – e.g. for healthcare staff who support people with a learning disability to manage their diabetes e.g. `Diabetes in Healthcare Training Course’ (Diabetes UK, 2022b) and `Safe Use of Insulin e-learning package’ (Elearning for Healthcare, 2022). Provide ongoing learning disability specific mandatory training for healthcare staff e.g. Oliver McGowan training (Department of Health and Social Care (DHSC), 2025a)

**Improve communications, interprofessional collaborations and shared expertise**. Improve communication and networks between healthcare professions from primary care and specialist learning disabilities services. Improve continuity of care and provide ongoing support. For example, Use an Open Communication Tool to identify barriers to effective communication (Lewer and Harding, 2013).

**Provide support** – e.g. the person with a learning disability may need practical support with their blood glucose monitoring and insulin administration

**Technology** – e.g., glucose monitoring systems can facilitate diabetes management and alert the person and / or their supporters to high or low blood glucose levels

**Provide ongoing support** – e.g. ensure the person knows who to contact for advice and support

**Address any social barriers** – e.g. make the healthcare building and rooms accessible and appropriate to those with physical mobility issues.

**Person-centred planning** – e.g. plan for and make reasonable adjustments tailored to the individual. For example, some individuals may have a mild-to-moderate learning disability whilst others may have a serious or profound learning disability. Some may have physical as well as learning disabilities. Everyone is different. Therefore, support needs should be adjusted meet their individual needs and to enable them to be as independent as possible in their diabetes self-management.

NHS England (2023b) introduced a **Reasonable Adjustment Digital Flag** into the NHS Spine in 2023 to enable health and social care staff to record, share and view details of Reasonable Adjustments across the NHS, wherever the person is seen and / or treated

Proactively **involve people with a learning disability in diabetes-related research**

## Key Messages

▪ Optimal diabetes care for people with a learning disability is limited by a lack of reasonable adjustments within healthcare systems and gaps in accessible diabetes education.
▪ Delivering high quality, person-centred diabetes care relies on meaningful reasonable adjustments, involvement of caregivers and support for self-management.
▪ Accessible diabetes education for people with a learning disability and their caregivers is essential to improve understanding of the condition and effective self-management.
▪ A range of resources are available to support individuals, caregivers and healthcare professionals with providing appropriate education and making reasonable adjustments in practice.
▪ Diabetes research should be inclusive of people with a learning disability, ensuring equal access to research opportunities, particularly in studies involving advanced diabetes technologies.
